# Supplementary material for: Changes in functional outcome after a first-time stroke: Data from a longitudinal study
Source: PLoS One. 2025 Aug 8;20(8):e0330086. doi: 10.1371/journal.pone.0330086 (PMC12333991; doi:10.1371/journal.pone.0330086)
Supplement: S1 Table — (DOCX) [file pone.0330086.s001.docx]

**S1 Table. Frequencies and percentages of modified Rankin scale (mRS) scores.**

|  |  | **Time** | **mRS = 0** | **mRS =1** | **mRS = 2** | **mRS = 3** | **mRS = 4** | **mRS = 5** |
| --- | --- | --- | --- | --- | --- | --- | --- | --- |
| **All** | |  | n (%) | n (%) | n (%) | n (%) | n (%) | n (%) |
|  |  | Discharge | 16 (10.3) | 38 (24.5) | 18 (11.6) | 20 (12.9) | 33 (21.3) | 30 (19.4) |
|  |  | 1-month | 43 (27.7) | 46 (29.7) | 35 (22.6) | 9 (5.8) | 12 (7.7) | 10 (6.5) |
|  |  | 3-month | 76 (49.0) | 37 (23.9) | 18 (11.6) | 6 (3.9) | 8 (5.2) | 8 (5.2) |
|  |  | 6-month | 95 (61.3) | 30 (19.4) | 8 (5.2) | 3 (1.9) | 9 (5.8) | 7 (4.5) |
| **Stroke subtypes** | |  |  |  |  |  |  |  |
|  | Ischemic stroke | |  |  |  |  |  |  |
|  |  | Discharge | 16 (11.5) | 35 (25.2) | 15 (10.8) | 20 (14.4) | 30 (21.6) | 23 (16.5) |
|  |  | 1-month | 41 (29.5) | 44 (31.7) | 29 (20.9) | 9 (6.5) | 8 (5.8) | 8 (5.8) |
|  |  | 3-month | 72 (51.8) | 33 (23.7) | 15 (10.8) | 5 (3.6) | 5 (3.6) | 7 (5.0) |
|  |  | 6-month | 89 (64.0) | 26 (18.7) | 6 (4.3) | 3 (2.2) | 6 (4.3) | 6 (4.3) |
|  | Hemorrhagic stroke | |  |  |  |  |  |  |
|  |  | Discharge | 0 (0.0) | 3 (18.8) | 3 (18.8) | 0 (0.0) | 3 (18.8) | 7 (43.8) |
|  |  | 1-month | 2 (12.5) | 2 (12.5) | 6 (37.5) | 0 (0.0) | 4 (25.0) | 2 (12.5) |
|  |  | 3-month | 4 (25.0) | 4 (25.0) | 3 (18.8) | 1 (6.3) | 3 (18.8) | 1 (6.3) |
|  |  | 6-month | 6 (37.5) | 4 (25.0) | 2 (12.5) | 0 (0.0) | 3 (18.8) | 1 (6.3) |
| **Stroke severity** | |  |  |  |  |  |  |  |
|  | No stroke symptoms (NIHSS 0) | | |  |  |  |  |  |
|  |  | Discharge | 15 (53.6) | 8 (28.6) | 1 (3.6) | 3 (10.7) | 0 (0.0) | 1 (3.6) |
|  |  | 1-month | 17 (60.7) | 9 (32.1) | 2 (7.1) | 0 (0.0) | 0 (0.0) | 0 (0.0) |
|  |  | 3-month | 26 (92.9) | 2 (7.1) | 0 (0.0) | 0 (0.0) | 0 (0.0) | 0 (0.0) |
|  |  | 6-month | 27 (96.4) | 1 (3.6) | 0 (0.0) | 0 (0.0) | 0 (0.0) | 0 (0.0) |
|  | Minor stroke (NIHSS 1-4) | | |  |  |  |  |  |
|  |  | Discharge | 1 (1.3) | 28 (35.4) | 12 (15.2) | 14 (17.7) | 19 (24.1) | 5 (6.3) |
|  |  | 1-month | 24 (30.4) | 31 (39.2) | 19 (24.1) | 4 (5.1) | 1 (1.3) | 0 (0.0) |
|  |  | 3-month | 40 (50.6) | 27 (34.2) | 8 (10.1) | 2 (2.5) | 1 (1.3) | 0 (0.0) |
|  |  | 6-month | 53 (67.1) | 21 (26.6) | 1 (1.3) | 2 (1.3) | 3 (1.3) | 0 (0.0) |
|  | Moderate stroke (NIHSS 5-15) | | |  |  |  |  |  |
|  |  | Discharge | 0 (0.0) | 2 (5.4) | 5 (13.5) | 3 (8.1) | 13 (35.1) | 14 (37.8) |
|  |  | 1-month | 2 (5.4) | 6 (16.2) | 13 (35.1) | 5 (13.5) | 7 (18.9) | 4 (10.8) |
|  |  | 3-month | 10 (27.0) | 8 (21.6) | 9 (24.3) | 3 (8.1) | 4 (10.8) | 3 (8.1) |
|  |  | 6-month | 15 (40.5) | 8 (21.6) | 5 (13.5) | 2 (5.4) | 4 (10.8) | 3 (8.1) |
|  | Severe stroke (NIHSS 16-42) | | |  |  |  |  |  |
|  |  | Discharge | 0 (0.0) | 0 (0.0) | 0 (0.0) | 0 (0.0) | 1 (9.1) | 10 (90.9) |
|  |  | 1-month | 0 (0.0) | 0 (0.0) | 1 (9.1) | 0 (0.0) | 4 (36.4) | 6 (54.5) |
|  |  | 3-month | 0 (0.0) | 0 (0.0) | 1 (9.1) | 1 (9.1) | 3 (27.3) | 5 (45.5) |
|  |  | 6-month | 0 (0.0) | 0 (0.0) | 2 (18.2) | 0 (0.0) | 4 (36.4) | 4 (36.4) |
