# Supplementary material for: Changes in functional outcome after a first-time stroke: Data from a longitudinal study
Source: PLoS One. 2025 Aug 8;20(8):e0330086. doi: 10.1371/journal.pone.0330086 (PMC12333991; doi:10.1371/journal.pone.0330086)
Supplement: Attachment — Submitted filename: PONE-D-24-52408.pdf [file pone.0330086.s002.pdf]

Reviewer's Recommendation and Comments for Manuscript Number PONE-D-24-52408

This cohort describes factors of changes in functional outcome after a first-time stroke. The results of this paper show lots of outstanding outcomes of functional recovery after stroke and discharge. Functional status recovery after an acute stroke varies significantly depending on factors such as the severity and type of stroke, the area of the brain affected, the individual's pre-stroke health, and the medical intervention such as medications and rehabilitation. Although I appreciate such an achievement, I still have three major questions and those questions that authors should reveal key points that are included in the method.

1. Location and size of the stroke in the brain imaging by CT, MRI, or angiography.
2. Timing of treatment such as thrombolysis with tPA or mechanical thrombectomy or supportive care only.
3. Any multidisciplinary rehabilitation including physical therapy, occupational therapy, and speech therapy.
