# Supplementary material for: Changes in functional outcome after a first-time stroke: Data from a longitudinal study
Source: PLoS One. 2025 Aug 8;20(8):e0330086. doi: 10.1371/journal.pone.0330086 (PMC12333991; doi:10.1371/journal.pone.0330086)
Supplement: Attachment — Submitted filename: Responses to reviwer comments-R1-PONE-D-24-52408(NB).docx [file pone.0330086.s004.docx]

**Response letter to the reviewer(s)' comments on manuscript ID PONE-D-24-52408**

**Title:** “Changes in functional outcome after a first-time stroke: Data from a longitudinal study”

Dear Editor,

Thank you for your comments and the reviewers' suggestions on our manuscript, "Changes in functional outcome after a first-time stroke: Data from a longitudinal study" (ID PONE-D-24-52408). All authors are very grateful for the constructive and valuable feedback from the two reviewers, which has significantly helped us improve this revised version of the manuscript.

Please find our point-by-point responses to the reviewers' comments on the following pages. In accordance with the instructions in your letter, we have uploaded a rebuttal letter that addresses each point raised by the academic editor and reviewers in the 'Response to Reviewers' section. Additionally, we have submitted a clean version of the revised manuscript under the 'Manuscript' section, along with a highlighted version as a separate file in the 'Revised Manuscript with Track Changes' section.

Furthermore, we have added more information to Figure 1, corrected a typo in Figure 2, and uploaded the new versions of both figures to the system. If you have any questions, please feel free to let me know.

Sincerely,

Nipaporn Butsing, RN, DrPH

Assistant Professor, Ramathibodi School of Nursing

Faculty of Medicine Ramathibodi Hospital, Mahidol University, Bangkok, Thailand.

Email: [nipaporn.but@mahidol.edu](mailto:nipaporn.but@mahidol.edu)

**A point-by-point response to the comments**

In the revised manuscript, we have made the following updates as suggested by the editor and the reviewers.

**Journal Requirements:**

**Comment 1:** Please ensure that your manuscript meets PLOS ONE's style requirements, including those for file naming. The PLOS ONE style templates can be found at

<https://journals.plos.org/plosone/s/file?id=wjVg/PLOSOne_formatting_sample_main_body.pdf> and

<https://journals.plos.org/plosone/s/file?id=ba62/PLOSOne_formatting_sample_title_authors_affiliations.pdf>

**[Authors’ Responses]:** We checked PLOS ONE's style templates for formatting guidelines for manuscript body, title, author, and affiliation. We ensure that the manuscript meets PLOS ONE's style requirements.

**Comment 2:** In the online submission form, you indicated that [Data cannot be shared publicly because of the institution's data-sharing policy, as it contains patients' clinical information. Data is available upon request and ethics committee approval. Researchers may submit their research proposals to the Human Research Ethics Committee, Faculty of Medicine Ramathibodi Hospital at https://www.rama.mahidol.ac.th or contact the corresponding author for help.].

All PLOS journals now require all data underlying the findings described in their manuscript to be freely available to other researchers, either 1. In a public repository, 2. Within the manuscript itself, or 3. Uploaded as supplementary information.

This policy applies to all data except where public deposition would breach compliance with the protocol approved by your research ethics board. If your data cannot be made publicly available for ethical or legal reasons (e.g., public availability would compromise patient privacy), please explain your reasons on resubmission and your exemption request will be escalated for approval.

**[Authors’ Responses]:** Our intuition policies permit data sharing only after a data sharing agreement has been signed by both parties—the data owner and the recipients—and after obtaining approval from the institution’s ethics committee. Therefore, we need to obtain ethics approval first. Once that is in place, we can share our study raw data with other researchers through a data sharing agreement. However, all relevant data are provided in the manuscript and the Supporting Information file.

**Reviewers' comments:**

**Reviewer's Responses to Questions**

**Comments to the Author**

1. Is the manuscript technically sound, and do the data support the conclusions?

The manuscript must describe a technically sound piece of scientific research with data that supports the conclusions. Experiments must have been conducted rigorously, with appropriate controls, replication, and sample sizes. The conclusions must be drawn appropriately based on the data presented.

Reviewer #1: Yes

Reviewer #2: Partly

2. Has the statistical analysis been performed appropriately and rigorously?

Reviewer #1: Yes

Reviewer #2: Yes

3. Have the authors made all data underlying the findings in their manuscript fully available?

The PLOS Data policy requires authors to make all data underlying the findings described in their manuscript fully available without restriction, with rare exception (please refer to the Data Availability Statement in the manuscript PDF file). The data should be provided as part of the manuscript or its supporting information, or deposited to a public repository. For example, in addition to summary statistics, the data points behind means, medians and variance measures should be available. If there are restrictions on publicly sharing data—e.g. participant privacy or use of data from a third party—those must be specified.

Reviewer #1: Yes

Reviewer #2: Yes

4. Is the manuscript presented in an intelligible fashion and written in standard English?

PLOS ONE does not copyedit accepted manuscripts, so the language in submitted articles must be clear, correct, and unambiguous. Any typographical or grammatical errors should be corrected at revision, so please note any specific errors here.

Reviewer #1: Yes

Reviewer #2: No

**5. Review Comments to the Author**

Please use the space provided to explain your answers to the questions above. You may also include additional comments for the author, including concerns about dual publication, research ethics, or publication ethics. (Please upload your review as an attachment if it exceeds 20,000 characters)

**Reviewer #1:**

This cohort describes factors of changes in functional outcome after a first-time stroke. The results of this paper show lots of outstanding outcomes of functional recovery after stroke and discharge. Functional status recovery after an acute stroke varies significantly depending on factors such as the severity and type of stroke, the area of the brain affected, the individual’s pre-stroke health, and the medical intervention such as medications and rehabilitation. Although I appreciate such an achievement, I still have three major questions and those questions that authors should reveal key points that are included in the method.

**Comment 1:** Location and size of the stroke in the brain imaging by CT, MRI, or angiography.

**[Authors’ Responses]:** Thank you for your valuable advice. The initial project did not record the size of the stroke; instead, we classified stroke severity based on the National Institutes of Health Stroke Scale (NIHSS) and the modified Rankin Scale (mRS) at discharge. This approach allowed us to focus on assessing the improvement in functional outcomes after treatment. However, we did collect information on the location of the stroke, which is presented in Table 1 and page 9; lines 190-193.

**Comment 2:** Timing of treatment such as thrombolysis with tPA or mechanical thrombectomy or supportive care only.

**[Authors’ Responses]:** Thank you for your valuable advice. The specific treatment for acute stroke based on timing has been added to Table 1.

**Comment 3:** Any multidisciplinary rehabilitation including physical therapy, occupational therapy, and speech therapy.

**[Authors’ Responses]:** Thank you for your valuable advice. Typically, multidisciplinary teams collaborate to treat stroke patients in this study setting. Teams specializing in physical, occupational, and speech therapy are consulted based on each patient's specific problems and needs. However, a limitation of our initial project is that we did not collect data on the consultations and rehabilitation programs each patient received. Therefore, we have included additional information regarding the rehabilitation program within this study setting (page 21; lines 354-360) and addressed this issue in the discussion section and included recommendations for future studies (page 22; lines 388-391).

**Reviewer #2:**

Authors report an interesting analysis of stroke outcome in Thailand, pointing out the profound differences between minor and severe strokes, the worse outcome of ICH and longer F.U. time needed for ICH, etc. However, some flaws arise, as follows.

**Comment 1:** The selection process is not clearly described. For example, at line 103 - “The primary study included 186 consecutive acute first-stroke patients hospitalized…” - the primary study is not cited and is not possible to piece together the origin of this study cohort.

**[Authors’ Responses]:** Thank you for your valuable feedback. The main project initially screened 186 consecutive patients who experienced their first stroke at an acute stroke unit from April 2020 to February 2021. Out of these, 155 patients met the inclusion criteria and were subsequently recruited for the study, which continued until six months after discharge (ending in August 2021). At the time of discharge, we collected data on the general characteristics, clinical characteristics, quality of life, and functional outcomes (measured by the Barthel Index and the Modified Rankin Scale) of all 155 eligible participants. Follow-up assessments of their quality of life and functional outcomes were conducted at one, three, and six months post-discharge. All data collection of the main project was approved by the Human Research Ethics Committee (Approval Number: COA. MURA2020/193). We have not reported all the findings in total, as the concept of quality of life is broad and relates to factors that differ from those influencing functional outcomes. Therefore, we decided to analyze and present the results for quality of life and functional outcomes separately. To pursue this, we applied the two subprojects to the Human Research Ethics Committee to get approval for each study to use the data from the main project. The ethical approval for the quality of life study is COA. MURA2024/48 (dated January 19, 2024), while the approval for the functional outcomes subproject is COA. MURA2024/597 (dated August 23, 2024). The data regarding changes in quality of life among the 155 study participants has already been reported in a previous study (Butsing N, Voss JG, Keandoungchun J, Thongniran N, Griffin MTQ. "Changes of health-related quality of life within 6 months after stroke by clinical and sociodemographic factors." Sci Rep. 2025 Jan 2;15(1):416. doi: 10.1038/s41598-024-84454-5. PMID: 39747957; PMCID: PMC11695920). In this recent study, we have cited the previous work and noted that some characteristics of the study samples were reported in earlier results (page 6; lines 117-118).

**Comment 2:** It also seems that Authors had “a priori” excluded fatal strokes and those patients who died later during the F.U. period (or mRS = 6), but this point not clear. I would be quite impressed that no death occurred at least within hemorrhagic and severe strokes. More, they state anyway that one patient died before 6 months F.U., and this contributes to make unclear how they managed fatal strokes. Overall, this point should be addressed/explained in M&M and in the “sample selection process flow chart (figure 1). It could be mentioned in the Discussion as well.

**[Authors’ Responses]:** Thank you for your valuable feedback. We reported that one patient died from sepsis during the follow-up period of one to three months (see page 7-8, lines 154-155), and this information has also been included in the sample selection flow chart (Figure 1). Additionally, we only recruited cognitively intact participants who could provide informed consent for the study. Since the follow-up period was limited to six months, there were no reported deaths due to stroke in our study. Therefore, we discussed that “this study only included cognitively intact patients. Those with more severe strokes, cognitive impairments, or communication difficulties due to aphasia were excluded, which may have led to an overestimation of functional outcomes.” (page 22; lines 382-384).

**Comment 3:** Furthermore, the exclusion of aphasic patients should be at least reported as a limitation since aphasia is one of the most important neurological deficits after stroke

**[Authors’ Responses]:** Thank you for your valuable suggestion. The limitation of excluding aphasic patients was discussed: “this study included only cognitively intact patients. Those with more severe strokes, cognitive impairments, or communication difficulties due to aphasia were excluded, which may have led to an overestimation of functional outcomes.” (page 22; lines 382-384).

**Comment 4:** Authors did not mention/consider rehabilitation regimens (i.e., intensive, extensive, outpatients’ programs, no rehab at all, etc.) in their analysis. At least the proportion of patients who underwent an active intervention to modify functional outcome could be reported.

**[Authors’ Responses]:** Thank you for your invaluable advice. We have included additional information regarding the rehabilitation program within this study setting (page 21; lines 354-360) and incorporated this topic into the discussion section and recommendations for future studies (page 22; lines 388-391).

**Comment 5:** The following two sentences (lines 232-235) are hard reading, and they seem contradictory, please clarify: “Participants with hemorrhagic stroke had poorer functional outcomes than those with ischemic stroke, with significant changes in mRS median scores from discharge to 1 month, 3 months, and 6 months post-discharge (p < 0.05). However, there was no significant change in mRS scores from 1-month to 3-month after discharge from hemorrhagic stroke”.

**[Authors’ Responses]:** Thank you for your valuable suggestion. We revised these two sentences to “Participants with hemorrhagic stroke showed poorer functional outcomes than those with ischemic stroke. There were significant changes in mRS median scores from discharge to 1 month, 3 months, and 6 months post-discharge among hemorrhagic stroke participants (p < 0.05), but no significant change from 1 month to 3 months after discharge.” (page 13; lines 232-236).

**Comment 6:** English phrasing must be revised in several sections. Following, only very few examples:
Line 25 - and varied by according to/depending on several factors…
Line 56 - worldwide the world [1].
Line 80 – 3 months…
Within Figure 1: all strokes

**[Authors’ Responses]:** Thank you for your valuable suggestion. English paraphrasing was corrected as suggested (page 2; line 25, page 3; line 53, page 4; line 77, page 6; line 116, page 9; line 195, page 12; line 213, page 13; lines 226-227, page 19; line 315, page 20; line 348, and the first picture in Figure 2).
